# Association between hypertension and dementia risk in low- and middle-income countries: A systematic review

**DOI:** 10.1016/j.jarlif.2025.100027

**Published:** 2025-09-06

**Authors:** Josephine E. Lindhout, Marieke P. Hoevenaar-Blom, Jan Willem van Dalen, Manshu Song, Dong Lin, Wei Wang, Edo Richard, Eric P. Moll van Charante, Tessa van Middelaar

**Affiliations:** a Department of Public and Occupational health, Amsterdam University Medical Centre, Amsterdam, The Netherlands; b Department of Neurology, Amsterdam University Medical Centre, Amsterdam, The Netherlands; c Department of General Practice, Amsterdam University Medical Centre, Amsterdam, The Netherlands; d Department of Neurology, Donders Institute for Brain, Cognition and Behaviour, Radboud University Medical Centre, Nijmegen, The Netherlands; e School of Medical and Health Sciences, Edith Cowan University, Perth, Australia; f Department of Cardiology, First Affiliated Hospital of Shantou University Medical College, Shantou, China; g Chemistry and Chemical Engineering Guangdong Laboratory, Shantou, China; h Institute for Glycome Study and The First Affiliated Hospital, Shantou University Medical College, Shantou, China; i School of Public Health, Shandong First Medical University & Shandong Academy of Medical Sciences, Jinan, China

**Keywords:** Dementia, Hypertension, Low- and middle income countries, Systematic review, Prevention

## Abstract

**Background:**

Dementia prevalence is rising most rapidly in low- and middle-income countries (LMICs), yet most evidence on risk factors such as hypertension stems from high-income settings. In LMICs, hypertension may have a greater impact due to its high prevalence and poor control. We systematically reviewed evidence on the association between blood pressure and dementia and cognition in LMICs, and compared findings across regions.

**Methods:**

We searched PubMed, Embase, PsycINFO, and Global Index Medicus and reviewed reference lists for relevant studies. We included longitudinal studies (follow-up ≥6 months) from LMICs on the association between systolic blood pressure or hypertension and incident dementia, mild cognitive impairment (MCI), or cognition, with a sample size of ≥500 individuals. Risk of bias was assessed using a modified Newcastle-Ottawa Scale.

**Results:**

Of 8709 screened articles, 26 were included: 19 from Asia, six from Latin America, and one from Africa. Operationalization of hypertension and cognitive outcome was heterogeneous across studies, ranging from using routine care data to triple blood pressure measurements and comprehensive cognitive screening with expert review and validation. Follow-up duration ranged from 7 months to 16 years. Hypertension was associated with a higher risk of incident dementia (RR 1.26, 95 %CI 1.03 – 1.53) and MCI (RR 1.19, 95 %CI 1.09 – 1.29). Due to limited number of studies per region, we were unable to compare effect sizes across geographical regions.

**Conclusion:**

Hypertension is associated with an increased risk of dementia and cognitive impairment in LMICs, but limited studies from Latin America and especially from Africa prevented reliable regional comparisons.

## Highlights

• In Low and Middle Income Countries (LMICs) hypertension is associated with an increased risk of mild cognitive impairment and dementia.
• Our review confirms known associations from High Income Countries, but adds contextual relevance for LMICs.
• The majority of the identified studies originate from China, and we identified very limited studies from Latin-America and Africa, which might limit generalizability of our findings.

## 1 Introduction

The global prevalence of dementia is projected to triple between 2019 and 2050, reaching up to 150 million cases [1] This rise is unevenly distributed, with the largest relative increase occurring in low- and middle-income countries (LMICs). In response, the scientific community has called for action through “The Nairobi Declaration on Reducing the Burden of Dementia in LMICs” [2], advocating for greater focus on underrepresented populations in dementia research. The strongest drivers of the growing number of dementia cases in LMICs include population ageing, population growth, and a higher burden of modifiable risk factors [1,3] High blood pressure, especially in midlife, is a well-established risk factor for cognitive decline and dementia [3] In recent decades, changes in diet and lifestyle have led to a rise in the burden of non-communicable diseases in LMICs, with high blood pressure currently being the leading risk factor for disease burden worldwide [4] However, awareness of hypertension in LMICs remains low, with only one-third-of individuals aware of their condition, and control rates as low as around 8 % [5] As a result, prevalence of hypertension is higher, making its population attributable fraction for dementia greater in LMICs than the global average (4–9.3 % vs 2 %) [6] Antihypertensive treatment presents a scalable and cost-effective opportunity for dementia prevention due to its simple, inexpensive, and widely implemented nature [7,8] Given the rapid aging of populations in LMICs [4], understanding the relationship between hypertension and dementia and cognitive decline in these settings is particularly relevant. However, most evidence originates from high-income countries (HICs) [9,10], raising concerns about its generalizability to LMICs.

Our primary aim is to systematically examine the association between hypertension and incident dementia in LMICs and to compare this association across geographical regions. Our secondary aim is to investigate the association of hypertension with mild cognitive impairment (MCI) and cognitive decline.

## 2 Methods

The protocol for this systematic review was registered on Prospero (ID: CRD42024568574) [11] The manuscript followed the *Preferred Reporting Items for Systematic Reviews and Meta-Analyses* (PRISMA) statement [12]

### 2.1 Literature searches

We searched PubMed, Embase, PsycINFO, and Global Index Medicus (a database with biomedical and public health literature from LMICs) on July 23, 2024. The search strategy consisted of three components: (1) dementia/MCI/cognition and (2) hypertension/blood pressure had to be included in the title or abstract, and (3) a LMIC had to be mentioned in the full text (Supplementary Methods 1–3: Complete search strategy). We also screened the reference lists of included articles and related systematic reviews to identify additional eligible studies. There were no language or date restrictions. Two authors (TvM and JL) independently screened potentially eligible studies using Rayyan[13] and EndNote [14], first based on titles and abstracts, then on full texts. In cases of disagreement, a third author MH-B was consulted.

### 2.2 Study selection

The following inclusion criteria were applied: (1) observational study with at least 6 months of follow-up; (2) conducted in LMICs, which we operationalized as being defined as Low, Lower-Middle or Upper-Middle Income Country by the World Bank at time of search in 2024[15]; (3) dementia, MCI, or cognitive functioning as a primary or secondary outcome; and (4) hypertension (defined by mean blood pressure, a clinical diagnosis of hypertension, and/or use of antihypertensive medications (AHM)) or blood pressure as an exposure. Studies were excluded if they had a sample size of fewer than 500 participants or were based on disease-specific cohorts (e.g., post-stroke or myocardial infarction).

### 2.3 Data extraction and risk of bias assessment

Data were extracted using a piloted data extraction form. The extracted information included: first author, year of publication, geographical region, study design, sample size, baseline characteristics of participants, definition of exposure and outcome, covariate adjustments, and effect sizes of the association between exposure (i.e., hypertension or blood pressure) and outcome (i.e., dementia, MCI, or cognition). Risk of bias (ROB) was assessed using the Newcastle-Ottawa Scale for non-randomised studies [16] This scale ranges from 0 to 9 and assesses the quality of a study based on three domains: selection, comparability, and outcome. We adapted the scale specifically for this context (Supplementary Table 1). A total score ≥ 7 was classified as low risk of bias, 4–6 as moderate risk, and ≤3 as high risk. Both data extraction and ROB assessment were conducted independently by one author (JL or MH-B) and checked by another author (TvM or JL). For two articles available only in Chinese, JL extracted data using ChatGPT (GPT-4o mini), and Copilot for translation, with verification by two native Chinese speakers (MS and DL) [17,18] If essential data were missing, corresponding authors were contacted for additional information.

### 2.4 Data synthesis

We performed meta-analyses to assess the association of hypertension with dementia, MCI, and cognitive decline. When possible, analyses were stratified by geographical region (Africa, Asia, Latin America). Data were pooled if there were ≥3 studies that analysed a comparable association. To enable pooling, odds ratios (ORs) were transformed to risk ratios (RRs) using the base-rate risk and the *effectsize* package in R [19] If the base-rate risk was unavailable for the analytical subsample, corresponding authors were contacted for additional information. Since event rates were low, hazard ratios (HRs) were considered equivalent to RRs to allow for pooling of the data [20] Effect sizes were pooled using a random-effects model (*meta* package in R version 4.3.2), as we anticipated considerable heterogeneity between studies. Between-study variance was estimated using the Der Simonian-Laird estimator, and confidence intervals were estimated based on standard normal quantiles [21] Heterogeneity between studies was assessed using the *I^2^* statistic, interpreted according to the Cochrane Handbook (<40 % might not be important, 30–60 % may represent moderate heterogeneity, 50–90 % substantial heterogeneity, and >75 % considerable heterogeneity) [22] Publication bias was assessed using a funnel plot if >10 studies were available for pooling [23] When pooling was not feasible, we conducted a narrative synthesis. For sensitivity analyses, we excluded studies with high or moderate risk of bias and separately analysed midlife vs. late-life hypertension as exposures.

## 3 Results

### 3.1 Selection of articles

**Fig. 1:**
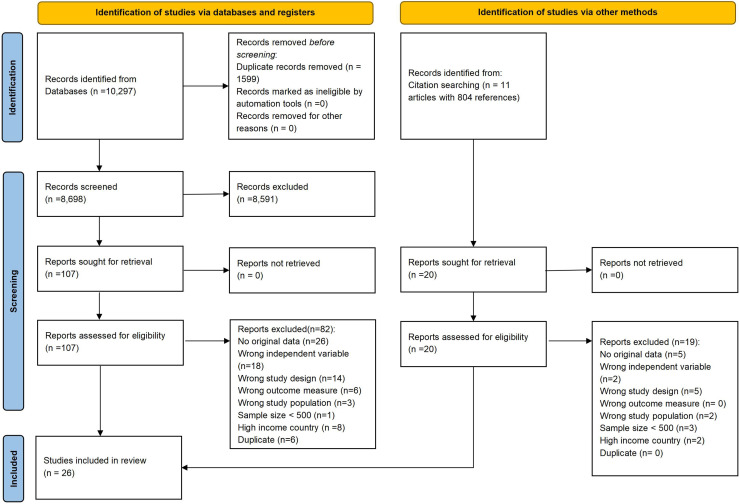
Flowchart screening.

We identified 10,297 articles from database searches. After deduplication, 8698 articles were screened based on title and abstract. Of these, 11 review articles were deemed potentially relevant, leading to citation searching of 804 references. In total, 127 articles underwent full-text review, of which 26 were included in this review reporting on 18 individual cohorts (Fig. 1).

### 3.2 Study characteristics

We identified longitudinal studies from eight countries across three geographical regions: 19 from Asia[[24], [25], [26], [27], [28], [29], [30], [31], [32], [33], [34], [35], [36], [37], [38], [39], [40], [41], [42]] (of which 16 from China) [25,26,28,[30], [31], [32], [33], [34], [35], [36], [37], [38], [39], [40], [41], [42]], 6 from Latin America [[43], [44], [45], [46], [47], [48]], and one from Africa [49] The studies were based on 18 different cohorts, since five cohorts published multiple articles with different objectives that met our inclusion criteria: the China Health and Retirement Longitudinal Study (CHARLS) [26,31,35,40], Chinese Longitudinal Healthy Longevity Survey (CLHLS) [25,38,39], China Health and Nutrition Survey (CHNS) [32,37], Brazilian Longitudinal Study of Adult Health (ELSA-Brazil) [43,44], and Mexican Health and Aging Study (MHAS) [46,47] Of the included studies, 23 were prospective cohort studies[[24], [25], [26], [27], [28], [29],[31], [32], [33], [34], [35], [36], [37], [38], [39], [40], [41], [42], [43], [44], [45], [46], [47], [48], [49]] and three were routine healthcare registry studies [24,29,30] The total sample size was 447,097 individuals (range: 602–206,073), with follow-up durations ranging from 7 months to 16 years. Since we included multiple studies with different objectives from the same cohort these are not all unique individuals. Most studies (*n* = 24) were performed in the general population [[24], [25], [26], [27], [28],[30], [31], [32], [33], [34], [35],[37], [38], [39], [40], [41], [42], [43], [44], [45], [46], [47], [48], [49]] The remaining studies focused on specific populations: nutritional intervention cohort (*n* = 1)[36] and tertiary care population (*n* = 1)[29] (Table 1).

**Table 1:** Characteristics of studies included in the systematic review ordered by geographic region and country.

Author (year)	Country	Study period	Study design*	Cohort†	Population	Sample size	Age (mean, SD)	Sex ( % women)	Follow-up (years)	Exposure‡	Outcome§
Africa											
Ogunniyi (2011)	Nigeria	1992–2007	P	IIDRP	General	1753	76 (5)	1210 (69 %)	6	1. Ht, 2. SBP x 10mmHg	Dementia
Asia											
Cheng (2021)	China	1998–2018	P	CLHLS	General	10,660	N/A	5755 (54 %)	6	Ht	Dementia
Ding (2023)	China	2011–2018	P	CHARLS	General	4824	68 (10)	2972 (62 %)	8	Ht	CD
Gao (2009)	China	2003–2007	P	N/A	General	1737	71 (5)	922 (53 %)	2	Ht	CD
Lee (2013)	China	2005–2011	R	EHC	General	1453	Median 73 (N/A)	913 (63 %)	6	Ht	CD
Li (2024)	China	2011–2018	P	CHARLS	General	11,671	59 (9)	6155 (53 %)	7	1. Ht, 2. SBP	CD
Qin (2016)	China	1989–2004	P	CHNS	General	976	63 (7)	508 (52 %)	5	Tertile of SBP	CD
Qu (2005)	China	1998–2001	P	N/A	General	2197	N/A	1640 (56 %)	3	Ht	1. Dementia, 2. AD and VD
Ren (2022)	China	2014–2018	P	SYS-AD	General	1843	71 (5)	1577 (57 %)	4	Ht	1. MCI, 2. Dementia
Su (2022)	China	2011–2018	P	CHARLS	General	6954	N/A	3243 (47 %)	8	Ht	CD
Wu (2003)	China	1984–2000	P	NIC	Nutrition study cohort	602	N/A	N/A	16	Ht	AD
Xu (2018)	China	1997–2006	P	CHNS	General	4847	64 (5)	N/A	N/A	Ht	CD
Yi (2024)	China	2014–2018	P	CLHLS	General	1132	78 (8)	498 (44 %)	5	SBP	MCI
Yuan (2019)	China	2000–2014	P	CLHLS	General	12,281	Median, IQR 81 (65–109)	6036 (49 %)	6	1. Ht, 2. SBP x 10 mmHg	MCI
Zhang (2004)	China	1993–1997	P	N/A	General	2079	69 (6)	1031 (50 %)	4	SBP x 30 mmHg	1. MCI, 2. CD
Zhang (2021)	China	2014–2016	P	N/A	General	7874	69 (7)	4081 (52 %)	2	SBP	CD
Zhang (2022)	China	2011–2018	P	CHARLS	General	8957	58 (8)	4619 (52 %)	8	Ht	CD
Farron (2020)	India	2016–2019	P	LASI-DAD	General	2874	69 (7)	1042 (36 %)	7 months	Ht	1. CD, 2. Dementia
Boongird (2020)	Thailand	2006–2012	R	HCUR	General	206,073	63 (10)	54 %	6	Ht	Dementia
Lawongsa (2024)	Thailand	2009–2023	R	PHD	Tertiary clinic	127,016	N/A	66,878 (53 %)	15	Ht	Dementia
Latin America											
de Menezes (2021)	Brazil	2008–2014	P	ELSA	General	7248	59 (6)	3898 (55 %)	4	Ht	CD
Ferreira (2023)	Brazil	2008–2019	P	ELSA	General	11,390	51 (9)	6333 (56 %)	8	1. SBP, 2. Ht	CD
Ribeiro (2024)	Brazil	2000–2015	P	SABE	General	1042	68 (0)	632 (61 %)	15	Ht	CD
Llibre-Rodriguez (2017)	Cuba	2003–2011	P	AAP	Primary care	2010	N/A	N/A	4	Ht	Dementia
Mejia-Arango (2011)	Mexico	2001–2003	P	MHAS	General	6846	N/A	N/A	2	Ht	1.Dementia, 2. MCI
Renteria (2022)	Mexico	2001–2016	P	MHAS	General	758	57 (4)	433 (57 %)	14	Ht	MCI

*Study design: *P*=prospective, *R*=retrospective. **†**Full cohort names: **AAP** – Aging & Alzheimer Project, **CHARLS** – China Health and Retirement Longitudinal Study, **CHNS** – China Health and Nutrition Survey, **CLHLS** – Chinese Longitudinal Healthy Longevity Survey, **EHC** – Elderly Health Centre, **ELSA**– Brazilian Longitudinal Study of Adult Health, **HCUR** – Health Checks Ubon Ratchathani, **IIDRP** – Indianapolis-Ibadan Dementia Research Project, **LASI-DAD** – Longitudinal Aging Study in India (Dementia sub-study), **MHAS** – Mexican Health and Aging Study. ‡Exposure: Ht=hypertension, SBP=systolic blood pressure, SBP x 10 mmHg = SBP ordinalized by 10 mmHg increment, SBP x 30 mmHg = SBP ordinalized by 30 mmHg increment. §Outcome: CD=cognitive decline, MCI=mild cognitive impairment, AD=Alzheimer’s disease, VD=vascular dementia.

### 3.3 Exposures

The exposure of interest was hypertension in 18 studies [[24], [25], [26], [27], [28], [29], [30],[33], [34], [35], [36], [37],^40^,^43^,[45], [46], [47], [48]], continuous blood pressure in four studies[32,38,41,42] and four studies analysed both hypertension and blood pressure [31,39,44,49] Most studies defined hypertension as systolic blood pressure (SBP) ≥140 mmHg and/or diastolic blood pressure (DBP) ≥90 mmHg [[26], [27], [28],^30^,^31^,^34^,^37^,^39^,^40^,[42], [43], [44],^49^] However, two studies used higher cut-offs of SBP ≥160 mmHg or DBP ≥95 mmHg and persistently high at 170 mmHg [25,36] Five studies measured hypertension [24,32,33,45,46], three studies relied on self-report [35,47,48], and one study used registry data (Table 1 and Supplementary Table 3) [29]

### 3.4 Outcomes

Five studies analysed multiple outcomes [26,27,33,34,46,48] Dementia was an outcome of interest in ten studies [24,25,27,29,33,34,36,45,46,49], MCI in four[38,39,46,47] and cognitive functioning was analysed in 14 studies (Table 1 and Supplementary Table 3) [[26], [27], [28],[30], [31], [32],^35^,^37^,[40], [41], [42], [43], [44],^48^] The methods for diagnosing dementia or MCI were heterogeneous across studies, ranging from self-reported dementia and routine health care data diagnosis to comprehensive assessments by expert diagnostic teams, followed by validation procedures. The Mini-Mental State Examination (MMSE), including the Chinese or an adjusted version, was the most commonly (*n* = 9) used cognitive screening tool [30,[33], [34], [35],^38^,^39^,^41^,^42^]

### 3.5 Risk of bias in included studies

The included studies scored between 3 and 9 points on the adjusted Newcastle-Ottawa Scale for nonrandomized studies (range 0–9), with higher scores indicating lower risk of bias. Twenty-one studies had low ROB [[25], [26], [27], [28],^30^,[32], [33], [34],[37], [38], [39], [40], [41], [42], [43], [44],[46], [47], [48], [49]], four studies had moderate ROB[24,35,36,45] and one study had high ROB[29] based on the Newcastle Ottawa Scale. The ROB assessment showed that many studies lacked an adequate description and or definition of exposure (*n* = 9)[24,29,32,33,36,[45], [46], [47], [48]] and sufficient details on the adequacy of follow-up (*n* = 11)[24,26,29,31,32,35,[37], [38], [39],^45^,^48^] (Supplementary Table 2).

### 3.6 Meta- analysis

We pooled the associations of hypertension with dementia (*n* = 7)[24,25,29,34,36,45,49] and MCI (*n* = 3) [34,39,47] The outcomes and exposures of the remaining studies were too heterogeneous to conduct a meta-analysis.

#### 3.6.1 Dementia

**Fig. 2:**
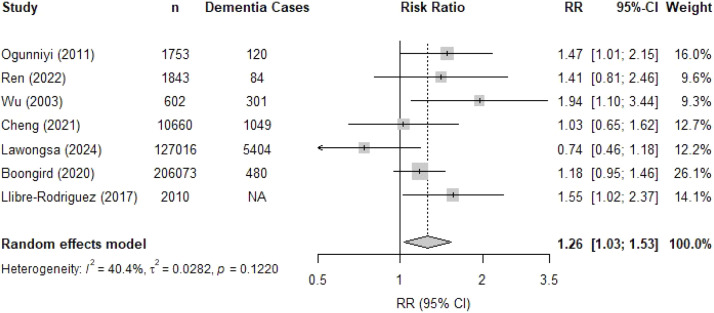
Forest plot of the association between hypertension and dementia.

**Fig. 3:**
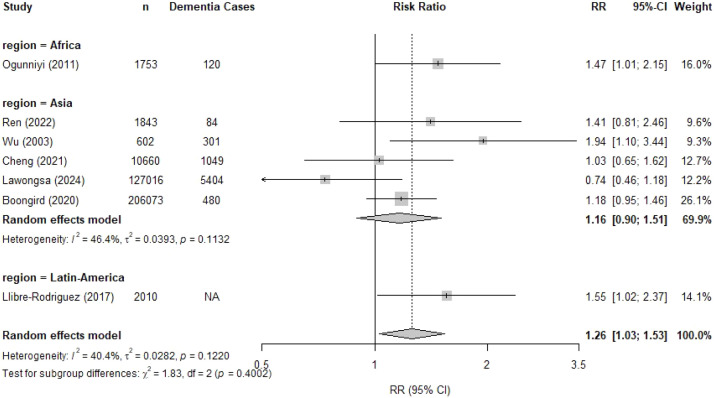
Forest plot of the association between hypertension and dementia pooled by geographic region.

Seven studies including a total of 349,957 individuals examined the relationship between hypertension and incident dementia, with a mean follow-up duration ranging from 4 to 16 years. Five studies were conducted in Asia [24,25,29,34,36], one in Latin America [45], and one in Africa [49] Hypertension was associated with a 26 % higher risk of developing dementia (Fig. 2; RR 1.26, 95 %CI 1.03 – 1.53). Heterogeneity was moderate with an *I^2^* of 40.4 %. For Asian studies, the pooled RR was 1.16 (95 %CI 0.90 – 1.51, 5 studies), compared to RR of 1.47 (95 % CI 1.01–2.15, 1 study) for the only African study in the meta-analysis and RR of 1.55 (95 %CI 1.02–2.37, 1 study) for the only Latin American study in the meta-analysis (Fig. 3). Excluding studies with moderate or high risk of bias, did not affect the results (RR 1.30, 95 %CI 1.00 – 1.68; Supplementary Figure 2).

#### 3.6.2 Mild cognitive impairment

**Fig. 4:**
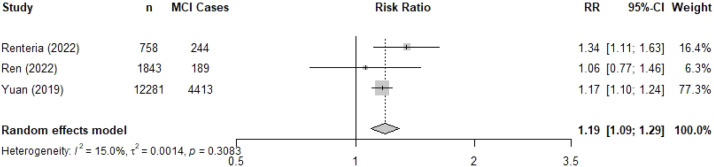
Forest plot of the association between hypertension and mild cognitive impairment.

Three studies including a total of 14,882 individuals reported the association between hypertension and incident MCI, with follow-up durations ranging from 4 to 14 years. The pooled analysis showed a 19 % increased risk of MCI in individuals with hypertension (RR 1.19, 95 %CI 1.09 – 1.29, *I^2^*: 15.0 %; Fig. 4). We did not pool results by geographic region due to the insufficient numbers of studies (two from Asia and one from Latin America).

### 3.7 Narrative synthesis of global cognitive score and cognitive domains

Three studies, including a total of 23,743 individuals, analysed the association between hypertension and global cognitive score, with follow-up durations ranging from 4 to 8 years [26,31,43] Eight studies, including a total of 51,650 individuals, analysed the association between hypertension and memory, most often assessed by delayed word recall [26,28,31,37,40,43,44] Seven studies found that hypertension was associated with lower memory scores after a median of 6 years (range: 2–8 years) and/or faster memory decline [26,28,31,37,40,43,44] One study showed no significant association (mean SBP divided in tertiles, highest vs lowest tertile β = 0.01 (95 %CI −0.03, 0.04) [32] Three studies found that hypertension was associated with lower executive functioning [26,43,44]

## 4 Discussion

### 4.1 Summary of main results

Hypertension is associated with an increased risk of MCI and dementia in LMICs, based on 19 Asian studies (of which 16 from China), six from Latin-America and one from Africa. Our findings confirm established associations in HICs and add important contextual relevance by demonstrating that the hypertension–dementia link also holds in LMIC settings. Although studies on cognitive domains were too heterogeneous for pooling, most suggested an association between hypertension and lower memory score and/or executive functioning. Geographic differences could not be reliably assessed due to the limited number of studies from Africa and Latin America. In regional analyses, no significant association was found in Asia, whereas significant associations were observed in Africa and Latin America. However, these latter findings were based on single studies only and should be interpreted with caution.

### 4.2 Evidence in context

The association between hypertension and dementia in this review (RR 1.26, 95 %CI 1.03 – 1.53) is comparable to the pooled results from predominantly HIC studies in a previous umbrella review (RR 1.20, 95 %CI 1.04 – 1.40) [50] This finding is particularly noteworthy, considering that several factors influencing the association between hypertension and dementia differ between LMICs and HICs. First, awareness and control of hypertension are significantly lower in LMICs [5,51] This may result in prolonged exposure to more severe hypertension, which could influence the observed association with dementia if the relationship is indeed causal. However, given the similar effect estimates to those from HICs, this influence may be less pronounced than expected. Due to the nature of the included studies, we were unable to adjust for duration and severity of hypertension. However, due to the nature of the included studies we were unable to adjust the analyses for duration of exposure to hypertension or to perform a meta-regression on the association between mean SBP and dementia to assess a dose-response relationship. Second, the higher prevalence of undiagnosed dementia in LMICs could introduce outcome misclassification, which in most cases would bias the association towards the null, potentially underestimating its true strength [51] Additionally, most research on the association between hypertension and dementia in HICs has been conducted primarily in White populations, limiting the generalizability of findings to ethnically diverse LMIC populations. It has been suggested that dementia risk and the impact of hypertension may vary across populations with different sociocultural backgrounds or genetic ancestry [52,53] Supporting this, several commonly used dementia risk prediction models—developed in HIC using combinations of weighted risk factors—performed inconsistently across LMICs, with accuracy varying significantly by country [54] This variability implies that the association between risk factors and dementia may not be uniform globally. Although our review was not specifically designed to examine ethnic or ancestral differences, it does highlight evidence from African, South Asian, and Latin American populations—groups that are often underrepresented in dementia research. Studying these populations within their local contexts is essential for deepening our global understanding of dementia risk factors and for developing more inclusive and effective prevention strategies. Hypertension prevention and management have proven cost-effective in HICs. Based on our findings showing that hypertension is associated to MCI and dementia and given the higher prevalence and lower control rates in LMICs, the potential impact of targeting this modifiable risk factor may be even greater in these settings [55] Effective prevention strategies in LMICs may include community health education, focused on diet and salt reduction, training primary care providers to follow hypertension treatment guidelines, and ensuring intensive follow-up for newly diagnosed patients. Equally important is the availability and affordability of essential medications [56]

While the projected increase in dementia is largest in LMICs, research on the association between blood pressure and dementia remains limited compared to HICs [1] Nonetheless, several initiatives are working to strengthen dementia research capacity in these underrepresented populations. The 10/66 research groups conducted surveys among older adults in ten LMICs revealing regional variations in the population attributable fractions (PAFs) of dementia risk factors. In line with our findings, the study reported a higher PAF for hypertension in Latin America (9 %) compared to Asia (4–6 %) [6] These PAFs primarily reflect the higher prevalence of hypertension in LMICs and are based on risk estimates derived from HIC data. If both the strength of the association and the prevalence of hypertension are greater in certain LMIC regions, actual PAFs could be even higher. Another important initiative, the Cohort Studies of Memory in an International Consortium (COSMIC), aims to harmonize data from global cohort studies—including both LMICs and HICs—on cognitive aging to support pooled analyses [57] Findings from COSMIC indicate that the association between the Lifestyle for BRAin Health (LIBRA) risk score and incident dementia is modified by geographic region, with a stronger association observed in Asian populations [58] Since risk prediction models, including LIBRA, are based on multiple weighted risk factors—not just hypertension—these results further underscore the importance of studying context and region-specific risk factor susceptibility.

In this review, several studies utilized routinely collected health care data [24,29,30] Using such data requires a routine health information system (RHIS), and sufficient high data quality. RHIS data are already widely used in sub-Saharan Africa to evaluate interventions for communicable diseases [59] Its application in dementia research could present both opportunities and challenges. On one hand, RHIS data allow long-term follow-up of large populations at relatively low cost. On the other hand, concerns remain about the validity of dementia diagnoses in RHIS data, particularly given the widespread underdiagnosis of dementia in the general population [60,61] A systematic review on the use of RHIS data for dementia research, which only included HIC-based studies, reported wide variation in diagnostic sensitivity (21–86 %) depending on the type and number of data sources used [62] To achieve the best results, a combination of primary care data (which offers high positive predictive value) alongside hospital and death registries (which contribute greater sensitivity) should be used. If data quality standards are met, RHIS data could complement prospective cohort studies in resource-limited settings by enabling extended follow-up, provide valuable insights into disease patterns and risk factors, and ultimately supporting the development of more effective prevention strategies.

### 4.3 Strengths and limitations

This systematic review has several strengths. We employed a comprehensive search strategy, searching four databases, including one with a special focus on studies from LMICs, and without language restrictions. The review comprised only studies with a sample size of at least 500 individuals and a follow up of at least 6 months to ensure the robustness of the evidence. The majority of the included studies had a low risk of bias (*n* = 21 out of 26). However, this review also has some important limitations. The availability of evidence was too sparse in some regions to answer whether there are differences between different geographical regions. The pooled effect size incorporates data from African, Latin American, and other Asian countries, but most studies included in this review are from China, which may introduce bias and limit generalizability. Although no language restrictions were applied to the full text, the search was conducted in English, so studies without English abstracts or keywords may have been missed. Heterogeneity in the definition of exposure (hypertension, SBP, AHM use) and outcome (dementia, MCI, cognitive decline, or decline in cognitive subdomains) hampered the pooling of results. Also the variation in definition of hypertension and dementia may lead to variation in validity of their diagnosis, leading to potential misclassification. For five studies hypertension was, solely or partially, based on self-report. We estimate that the influence of self-reported hypertension on the outcome is limited, because self-reported hypertension is probably a reliable proxy for objective hypertension [63] Diagnosis of dementia was mostly based on DSM-IV criteria (*n* = 4), two studies used a combination of cognitive screening test combined with neurologic examination or neuropsychologic testing, one study used self- or proxy reported diagnosis, one used registration data with ICD-10 or prescription data, and one based a cut-off on cognitive screening tests. Self- or proxy-reported hospital diagnosis could lead to underdiagnosis of dementia. Variability in cultural perceptions of illness, healthcare-seeking behavior, access to medical services, diagnostic criteria, and the organization of health information systems can all affect how frequently hypertension and dementia are diagnosed and recorded in medical registries. Consequently, cross-regional comparisons based on registry data could be influenced by these regional differences. We reported on the fullest model reported in each study and all studies at least adjusted for the three most important confounders; age, sex and education. Additional adjustments for other potential confounders varied significantly across studies. Lack of adjustment for other variables such as other vascular risk factors or medication adherence could lead to residual confounding. Finally, due to the limited number of studies specifically focusing on mid- or late life, or stratifying results by age category, we were unable to aggregate evidence by age group—despite the fact that the relationship between hypertension and dementia is known to vary across the lifespan [9,50]

## 5 Conclusion

Hypertension is associated with an increased risk of MCI and dementia in LMICs, comparable to associations found in HIC populations. This conclusion is largely based on studies from Asia, particularly China. While estimates from Africa and Latin America appear to be a bit higher, paucity of data from these regions precludes definitive conclusions about potential geographical differences.

## CRediT authorship contribution statement

**Josephine E. Lindhout:** Writing – original draft, Visualization, Methodology, Investigation, Formal analysis, Data curation, Conceptualization. **Marieke P. Hoevenaar-Blom:** Writing – review & editing, Supervision, Project administration, Methodology, Data curation, Conceptualization. **Jan Willem van Dalen:** Writing – review & editing, Methodology. **Manshu Song:** Writing – review & editing, Validation, Data curation. **Dong Lin:** Writing – review & editing, Validation, Data curation. **Wei Wang:** Writing – review & editing. **Edo Richard:** Writing – review & editing, Supervision, Project administration, Conceptualization. **Eric P. Moll van Charante:** Writing – review & editing, Writing – original draft, Supervision, Conceptualization. **Tessa van Middelaar:** Writing – review & editing, Writing – original draft, Supervision, Project administration, Methodology, Investigation, Funding acquisition, Formal analysis, Data curation, Conceptualization.

## Declaration of competing interest

The authors declare that they have no known competing financial interests or personal relationships that could have appeared to influence the work reported in this paper.

## Appendix Supplementary materials

Image, application 1
