# Supplementary material for: Association between hypertension and dementia risk in low- and middle-income countries: A systematic review
Source: JAR Life. 2025 Sep 6;14:100027. doi: 10.1016/j.jarlif.2025.100027 (PMC12446671; doi:10.1016/j.jarlif.2025.100027)

Appendix

Supplementary Text 1: Full search PubMed, Embase, PsycINFO

Supplementary Text 2: Full search Global Medicus

Supplementary Table 1: Operationalization of Newcastle-Ottawa-Scale for this review

Supplementary Figure 1: Forest plot of the association between hypertension and dementia pooled by age category of the exposure.

Supplementary Figure 2: Forest plot of the association between hypertension and dementia pooled by risk of bias rating.

## Supplementary Text 1: Full search PubMed, Embase, PsycINFO

(Dementia.sh OR dement*.ti OR dement*.ab OR Alzheimer*.ti OR Alzheimer*.ab OR cognition.sh OR cognit*.ti OR cognit*.ab OR Memory.sh OR memory.ti OR memory.ab)

AND

(Hypertension.sh OR hypertens*.ti OR hypertens*.ab OR (blood pressure).sh OR (blood pressure).ti OR (blood pressure).ab OR (Antihypertensive Agents).sh OR antihypertensive.ti OR antihypertensive.ab OR anti-hypertensive.ti OR anti-hypertensive.ab)

AND

((Developing Countries.sh) OR (low income countr*.ti) OR (low income countr*.ab) OR (low-income countr*.ti) OR (low-income countr*.ab) OR (middle income countr*.ti) OR (middle income countr*.ab) OR (middle-income countr*.ti) OR (middle-income countr*.ab) OR LMIC.ti OR LMIC.ab OR

Africa.sh OR Afric*.ti OR Afric*.ab OR (Central America.sh) OR (Central Americ*.ti) OR (Central Americ*.ab) OR (Latin America.sh) OR latin*.ti OR latin*.ab OR (South America.sh) OR (south americ*.ti) OR (south americ*.ab) OR Asia.sh OR Asia*.ti OR Asia*.ab OR Arabs.sh OR Arab*.ti OR Arab*.ab OR

Afghan* OR (Burkina Faso) OR Burundi* OR (Central African Republic) OR Chad* OR Congo* OR Kongo* OR Eritrea* OR Ethiopia* OR Gambia* OR Guinea* OR Korea* OR Liberia* OR Madagas* OR Malawi* OR Mali* OR Mozambi* OR Niger* OR Rwanda* OR (Sierra Leone*) OR Somali* OR Sudan* OR Syria* OR Togo* OR Uganda* OR Yemen* OR Zambia* OR

Angola* OR Algeria* OR Bangladesh* OR Benin* OR Bhutan* OR Bolivia* OR (Cabo Verde*) OR Cambodia* OR Cameroon* OR Comoros OR comorians OR d’Ivoire OR ivor* OR Djibouti* OR Egypt* OR Salvador* OR Eswatini OR swazi* OR Ghana* OR Haiti* OR Hondura* OR India* OR Indonesia* OR Iran* OR Kenya* OR Kiribati OR Kyrgyz* OR (Lao PDR) OR (Lao people*) OR Laos OR Leban* OR Lesotho OR Mauritania* OR Micronesia* OR Mongolia* OR Morocc* OR Myanmar OR Burm* OR Nepal* OR Nicaragua* OR Nigeria* OR Pakistan* OR Papua* OR Philip* OR Samoa* OR Tome OR Senegal* OR Solomon* OR Lanka* OR Tanzania* OR Tajik* OR Timor* OR Tunisia* OR Ukrain* OR Uzbek* OR Vanuatu OR Vietnam* OR Gaza OR Zimbabwe* OR

Albania* OR Argentin* OR Armenia* OR Azerbaijan* OR Belarus* OR Belize* OR Bosnia* OR Botswana* OR Brazil* OR Bulgaria* OR China OR Chinese OR Colombia* OR (Costa Rica*) OR Cuba* OR Dominica* OR Ecuador* OR Fiji* OR Gabon OR Georgia* OR Grenada OR Guatemala* OR Guyan* OR Iraq* OR Jamaica* OR Jordan* OR Kazakh* OR Kosov* OR Libya* OR Malaysia* OR Maldiv* OR Marshall* OR Mauriti* OR Mexic* OR Moldova* OR Montenegr* OR Namibia* OR Macedonia* OR Palau* OR Paraguay* OR Peru* OR Russia* OR Serb* OR Lucia OR Grenadines OR Surinam* OR Thai* OR Tonga* OR Turk* OR Tuvalu*)

## Supplementary Text 2: Full search Global Medicus

(ti:dement* OR ab:dement* OR ti:Alzheimer* OR ab:Alzheimer* OR ti:cognit* OR ab:cognit* OR ti:memory OR ab:memory)

AND

(ti:hypertens* OR ab:hypertens* OR ti:(blood pressure) OR ab:(blood pressure))

AND

(ti:(low income countr*) OR ab:(low income countr*) OR ti:(low-income countr*) OR ab:(low-income countr*) OR ti:(middle income countr*) OR ab:(middle income countr*) OR ti:(middle-income countr*) OR ab:(middle-income countr*) OR ti:LMIC OR ab:LMIC OR

ti:Afric* OR ab:Afric* OR ti:(Central Americ*) OR ab:(Central Americ*) OR ti:latin* OR ab:latin* OR ti:(south americ*) OR ab:(south americ*) OR ti:Asia* OR ab:Asia* OR ti:Arab* OR ab:Arab* OR

Afghan* OR (Burkina Faso) OR Burundi* OR (Central African Republic) OR Chad* OR Congo* OR Kongo* OR Eritrea* OR Ethiopia* OR Gambia* OR Guinea* OR Korea* OR Liberia* OR Madagas* OR Malawi* OR Mali* OR Mozambi* OR Niger* OR Rwanda* OR (Sierra Leone*) OR Somali* OR Sudan* OR Syria* OR Togo* OR Uganda* OR Yemen* OR Zambia* OR

Angola* OR Algeria* OR Bangladesh* OR Benin* OR Bhutan* OR Bolivia* OR (Cabo Verde*) OR Cambodia* OR Cameroon* OR Comoros OR Comorian OR d'Ivoire OR ivor* OR Djibouti* OR Egypt* OR Salvador* OR Eswatini OR swazi* OR Ghana* OR Haiti* OR Hondura* OR India* OR Indonesia* OR Iran* OR Kenya* OR Kiribati OR Kyrgyz* OR (Lao PDR) OR (Lao people*) OR Laos OR Leban* OR Lesotho OR Mauritania* OR Micronesia* OR Mongolia* OR Morocc* OR Myanmar OR Burm* OR Nepal* OR Nicaragua* OR Nigeria* OR Pakistan* OR Papua* OR Philip* OR Samoa* OR Tome OR Senegal* OR Solomon* OR Lanka* OR Tanzania* OR Tajik* OR Timor* OR Tunisia* OR Ukrain* OR Uzbek* OR Vanuatu OR Vietnam* OR Gaza OR Zimbabwe* OR

Albania* OR Argentin* OR Armenia* OR Azerbaijan* OR Belarus* OR Belize* OR Bosnia* OR Botswana* OR Brazil* OR Bulgaria* OR Chin* OR Colombia* OR (Costa Rica*) OR Cuba* OR Dominica* OR Ecuador* OR Fiji* OR Gabon OR Georgia* OR Grenada OR Guatemala* OR Guyan* OR Iraq* OR Jamaica* OR Jordan* OR Kazakh* OR Kosov* OR Libya* OR Malaysia* OR Maldiv* OR Marshall* OR Mauriti* OR Mexic* OR Moldova* OR Montenegr* OR Namibia* OR Macedonia* OR Palau* OR Paraguay* OR Peru* OR Russia* OR Serb* OR Lucia OR Grenadines OR Surinam* OR Thai* OR Tonga* OR Turk* OR Tuvalu*)

## Supplementary Table 1: Operationalization of Newcastle-Ottawa-Scale for this review

|  | *Item* | **Operationalization** |
| --- | --- | --- |
| *Selection* | *1. Representativeness of the exposed cohort* | **Truly or somewhat representative of the average older adult without dementia in the community (*/-)** |
|  | *2. Selection of the -n-exposed cohort* | **Drawn from the same community as the exposed cohort** |
|  | *3. Ascertainment of exposure (hypertension/systolic BP)* | **Continuous or categorized by established cut-offs** |
|  | *4. Demonstration that outcome of interest was present at start of study* | **yes/no** |
| *Comparability* | *Study controls for* | **Age** |
|  | *Study controls for* | **Sex** |
|  | *Study controls for* | **Education** |
|  | *Total comparability score* | ***=controls for age, **= controls for age, sex and education, -=crude only** |
| *Outcome* | *1. Ascertainment of outcome* | **Independent assessment (NPO/MMSE/other validated cognitive test)** |
|  | *2. Was follow-up long enough for outcomes to occur?* | **≥ 1 year** |
|  | *3. Adequacy of follow up of cohorts* | **Adequate description or > 80%** |
|  | *Total score (max. 9)* | **0-9** |

##

| **Study** | **Exposed cohort relevance** | **Non-exposed selection** | **Exposure check** | **Outcome absent at baseline** | **Comparability score** | **Outcome verification** | **Duration of follow-up** | **Adequacy of follow-up** | **Quality score (0-9)** |
| --- | --- | --- | --- | --- | --- | --- | --- | --- | --- |
| Ogunniyi 2011 | * | * | * | * | ** | * | * | * | 9 |
| Cheng 2021 | * | * | * | * | ** | - | * | * | 8 |
| Ding 2022 | * | * | * | * | ** | * | * | - | 8 |
| Gao 2009 | * | * | * | * | ** | * | * | * | 9 |
| Lee 2013 | * | * | * | * | * | * | * | * | 8 |
| Li 2024 | * | * | * | * | ** | * | * | - | 8 |
| Qin 2016 | * | * | - | * | ** | * | * | - | 7 |
| Qu 2005 | * | * | - | * | ** | * | * | * | 8 |
| Ren 2022 | * | * | * | * | ** | * | * | * | 9 |
| Su 2022 | * | * | * | * | - | * | * | - | 6 |
| Wu 2003 | * | * | - | - | - | * | * | * | 5 |
| Xu 2018 | * | * | * | * | ** | * | * | - | 8 |
| Yi 2024 | * | * | * | * | ** | * | * | - | 8 |
| Yuan 2019 | * | * | * | * | ** | * | * | - | 8 |
| Zhang 2004 | * | * | * | * | ** | * | * | * | 9 |
| Zhang 2021 | * | * | * | * | ** | * | * | * | 9 |
| Zhang 2022 | * | * | * | * | ** | * | * | * | 9 |
| Farron 2020 | * | * | * | * | ** | * | - | * | 8 |
| Boongird 2020 | * | * | - | * | * | - | * | - | 5 |
| Lawongsa 2024 | - | - | - | * | * | - | * | - | 3 |
| de Menezes 2020 | * | * | * | * | ** | * | * | * | 9 |
| Ferreira 2023 | * | * | * | * | ** | * | * | * | 9 |
| Ribeiro 2024 | * | * | - | * | ** | * | * | - | 7 |
| Llibre-Rodriquez 2017 | * | * | - | * | - | * | * | - | 5 |
| Mejia-Arango 2011 | * | * | - | * | ** | * | * | * | 8 |
| Renteria 2022 | * | * | - | - | ** | * | * | * | 7 |

Supplementary Table 2: Risk of bias assessment according to the Newcastle-Ottawa Scale of studies included in the systematic review.

Supplementary Table 3: operationalization of exposure and outcome

| **Author (year)** | **Exposure*** | **Operationalization†** | **Outcome‡** | **Operationalization§** |
| --- | --- | --- | --- | --- |
| **Africa** | | | | |
| Ogunniyi (2011) | 1. Ht, 2. SBP x 10mmHg | 1. SBP≥140 mmHg, DBP≥90 mmHg (mean of 3 measurements), 2. SBP x 10mmHg. | Dementia | 1. CSI-D + Informant Score. 2. clinician home-based interview, neurologic & physical examination + structured interview of a relative. |
| **Asia** | | | | |
| Cheng (2021) | Ht | Persistantly high at 170 mmHg. | Dementia | Self- or proxy-reported hospital diagnosis. |
| Ding (2023) | Ht | SBP≥140 mmHg, DBP≥90 mmHg or use of AHM (including traditional Chinese herbal medicine). | CD | Global cogniton z-score |
| Gao (2009) | Ht | SBP≥140 mmHg, DBP≥90 mmHg (mean of 2 measurements), or self-reported. | CD | Decline on CERAD word list learning and word list recall. |
| Lee (2013) | Ht | SBP≥140 mmHg or DBP≥90 mmHg. | CD | Dementia, scoring below the cutoff point on C-MMSE, and a global CDR of 1–3. |
| Li (2024) | 1. Ht, 2. SBP | 1. SBP≥140 mmHg, DBP≥90 mmHg or use of AHM, 2. SBP x 10mmHg. | CD | Global cogniton z-score |
| Qin (2016) | Tertile of SBP | N/A | CD | ∆ in TICS-m |
| Qu (2005) | Ht | N/A | 1. Dementia, 2. AD & VD | 1. MMSE screening, 2. NPT for those with a lower MMSE and those with self-reported memory decline but without a lower MMSE, 3. Suspected cases were re-evaluated 6 months after the initial assessment. |
| Ren (2022) | Ht | SBP≥140 mmHg, DBP≥90 mmHg or use of AHM. | 1. MCI, 2. Dementia | 1. a. subjective cognitive complaints, defined as self-rated AD8 score≥2; and b. objective impairment in global cognition, defined as the MMSE score≥1 standard deviation (SD) below the age- and education-specific mean MMSE scores. 2. According to DSM-IV by trained neurologists based on clinical examinations, assessment of cognitive and physical functioning using structured questionnaires. |
| Su (2022) | Ht | Self-reported | CD | ∆ C-MMSE |
| Wu (2003) | Ht | SBP ≥160 mmHg or DBP ≥95 mmHg. | AD | DSM-IV criteria, based on clinical data reviewed by diagnostic team. |
| Xu (2018) | Ht | SBP≥140 mmHg, DBP≥90 mmHg or use of AHM. | CD | TICS-m |
| Yi (2024) | SBP | SBP continuous (mean of two measurements) | MCI | C-MMSE. Normal cognition 25-30, Mild CI 18-24, Moderate CI 10-17, Severe CI 0-9. |
| Yuan (2019) | 1. Ht, 2. SBP x 10 mmHg | SBP≥140 mmHg or DBP≥90 mmHg | MCI | MMSE < 24 |
| Zhang (2004) | SBP x 30 mmHg | SBP≥140 mmHg, DBP≥90 mmHg or use of AHM | 1. MCI, 2. CD | 1. Adjusted MMSE scores: Illiterate: ≤17, Primary school: ≤20, Middle school: ≤22, University: ≤24, 2. Δ 4 MMSE points |
| Zhang (2021) | SBP | SBP continuous (mean of two measurements). | CD | ∆ MMSE |
| Zhang (2022) | Ht | SBP≥140 mmHg, DBP≥90 mmHg or use of AHM. | CD | Total score of word recall test, TICS and redrawing picture. |
| Farron (2020) | Ht | SBP≥140 mmHg, DBP≥90 mmHg, or self-reported | 1. CD, 2. Dementia | 1. A summary cognition score, derived from the sum of 18 cognitive tests. |
| Boongird (2020) | Ht | N/A | Dementia | Physician diagnosed dementia according to DSM-IV in medical record |
| Lawongsa (2024) | Ht | ICD-10: I10-I15 | Dementia | ICD-10 code of F00-F03, G30, or a prescription for a cholinesterase inhibitor or an N-methyl-D-aspartate receptor antagonist. |
| **Latin America** | | | | |
| de Menezes (2021) | Ht | SBP≥140 mmHg, DBP≥90 mmHg or use of AHM | CD | Global cogniton z-score |
| Ferreira (2023) | 1. SBP, 2. Ht | 1. SBP continuous, 2. SBP≥140 mmHg or DBP≥90 mmHg | CD | Global cogniton z-score |
| Ribeiro (2024) | Ht | Self-reported | CD | 1. scoring ≤12/19 on abbreviated MMSE 2. ∆ abbreviated MMSE |
| Llibre-Rodriguez (2017) | Ht | N/A | Dementia | DSM-IV |
| Mejia-Arango (2011) | Ht | N/A | 1.Dementia, 2. MCI | 1. DSM-IV by a group of Geriatricians and Neuropsychologists, 2. Cognitive, but no functional impairment on the CCCE |
| Renteria (2022) | Ht | Self-reported | MCI | Composite cognitive domain score ≥1.5 SD below demographically corrected T-scores |

*****Exposure: Ht=hypertension, SBP=systolic blood pressure, SBP x 10 mmHg = SBP ordinalized by 10 mmHg increment, SBP x 30 mmHg = SBP ordinalized by 30 mmHg increment. †Operationalization exposure: SBP=systolic blood pressure, DBP= diastolic blood pressure, AHM= antihypertensive medication, ICD-10= international classification of disease, N/A= not available. §Operationalization outcome: CSI-D= community screening interview for dementia, CERAD= Consortium to Establish a Registry for Alzheimer’s Disease, C-MMSE= Chinese mini mental state examination, CDR= Clinical Dementia Rating, TICS-m= Modified Telephone Interview for Cognitive Status, NPT= Neuropsychological testing, DSM-IV= Diagnostic and Statistical Manual of Mental Disorders, fourth version, MMSE= Mini Mental State Examination, CCCE= Cross Cultural Cognitive Examination. ‡Outcome: CD=cognitive decline, MCI=mild cognitive impairment, AD=Alzheimer’s disease, VD=vascular dementia.

## Supplementary Figure 1: Forest plot of the association between hypertension and dementia pooled by age category of the exposure.


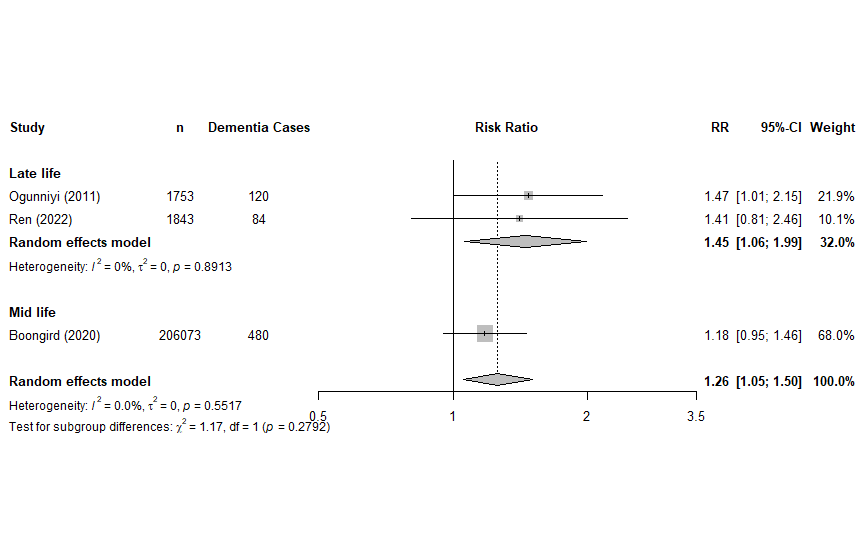


Supplementary Figure 2: Forest plot of the association between hypertension and dementia pooled by risk of bias rating.


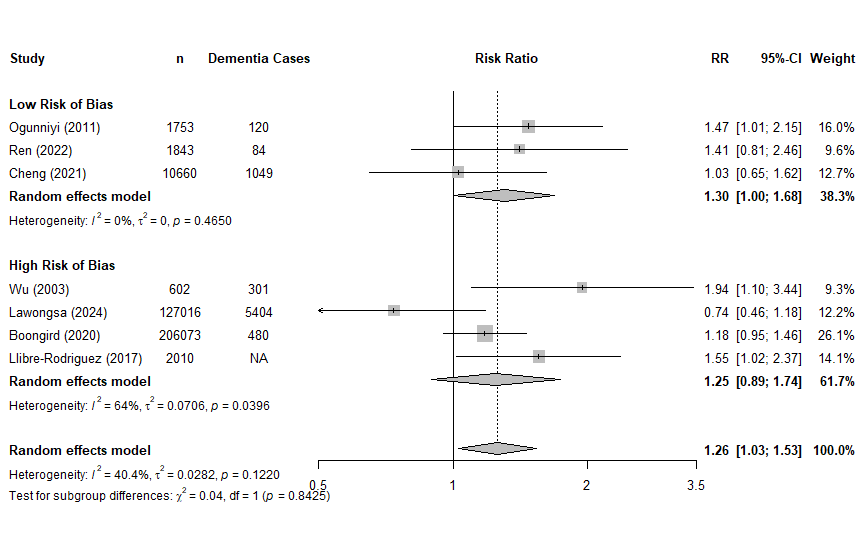

Supplement: Supplementary file 1 [file mmc1.docx]
